# Supplementary material for: Influenza vaccination hesitancy in large urban centers in South America. Qualitative analysis of confidence, complacency and convenience across risk groups
Source: PLoS One. 2021 Aug 12;16(8):e0256040. doi: 10.1371/journal.pone.0256040 (PMC8360613; doi:10.1371/journal.pone.0256040)
Supplement: S1 File — (DOCX) [file pone.0256040.s001.docx]

**SOCIAL DETERMINANTS OF INFLUENZA VACCINATION COVERAGE IN SELECTED COUNTRIES OF LATIN AMERICA**

**Focus group guide**

**Mothers of children under 5 years of age, pregnant women, adults with risk factors, and older adults**

The focus groups will discuss knowledge, attitudes and practices regarding prevention and care of influenza.

The following guide was developed for the application of focus groups with risk group participants. It is a thematic guide that should be flexibly applied to attain study objectives.

Study objective: Identify the knowledge, attitudes and practices about influenza and the influenza vaccine in the population of interest.

**Introduction**

- Registration of participants (see data sheet below)
- Personal presentation and thanks to participants
- Explain the group's objective and participation rules (speak in turn, be respectful of the opinions of others, do not talk about things that they would not like to be known outside the group, let others talk, do not use the cell phone during the session unless it is urgent; if necessary please do it outside)
- Request verbal informed consent and guarantee of confidentiality and use of information only for research purposes

**Presentation dynamics**

To begin, could you tell me your name, what do you do and something you enjoy doing? (It can be a hobby, some activity).

- Introductory question: Has anyone gotten sick or know someone who has gotten sick with the flu?

**Knowledge**

This section will explore what information particpants have about the disease, ways to prevent it, and the usefulness of the vaccine.

What is influenza?

How do you get the influenza?

What are the symptoms of the influenza? How can we know that we have influenza?

How can we avoid getting the influenza?

Where did you get this information from, who gave it to you? (explore if you have obtained it from a vaccination campaign)

Is it possible that medical personnel can do something to cure influenza?

What can you do if you get influenza?

What health services are there in your community to treat this disease?

What do you know about the influenza vaccine? (specifically explore utility and possible effects)

**Attitudes**

Attitudes express a position in front of an object that can potentially lead to a particular behavior. Attitudes express dispositions to act and that is what is explored in this section in relation to influenza and vaccination.

Do you consider influenza to be a preventable disease?

How? What can you do?

How important is it to you to know about influenza?

Why?

Do you think the information available on influenza is sufficient, useful, is it clear?

What do you think about this disease?

It's bad? Is it curable? Is it painful?

Do you think that at some point you can get the disease?

Who can get this disease? Why?

How easy is it to get an influenza vaccine shot?

Where can you be vaccinated?

Do you consider that the vaccine is reliable? Why?

Does the influenza vaccine have side effects?

As which? What do you think of these effects?

Can the influenza vaccine cause a reaction?

How is this reaction manifested? What do you think of this possibility?

Have you been vaccinated or would you be vaccinated? Why?

**Practices**

This section explores the actions that people carry out, especially focused on the prevention and care of influenza.

How do you take care of yourself not to get sick from influenza?

What prevention measures do they take?

If you have already had influenza, what have you done to make yourself feel better?

How have you been cared for?

In your homes, who decides what to do to avoid getting sick?

In your homes, who decides what care the family member who has fallen ill with influenza should have?

What does this person take into account when deciding what to do?

If this has been the case, what has made this person go to get vaccinated against influenza?

If this has been the case, what has made this person not go to get vaccinated against influenza?

If you have decided to get vaccinated, do you know where to go? Do you know when to go?

Are there people [other than medical personnel] in the community who treat influenza?

Who is it? How do they do that? Do you go with them?

**Closing**

Ask participants to describe what they actually did in the last vaccination campaign and from that description identify: the role of the context, individual and group influences and specific aspects of the vaccine.

Explore: What happened? (they were vaccinated / not vaccinated); Who told them to get vaccinated?; When did they go and who accompanied them ?; What was the result?; How did they feel?; Was it expensive ?; How were they treated in the health service?

**Participant registration**

| N° | Sex | Age | Education level | Occupation | Number of children | Social security affiliation or service provider |
| --- | --- | --- | --- | --- | --- | --- |
|  |  |  |  |  |  |  |
|  |  |  |  |  |  |  |
|  |  |  |  |  |  |  |
|  |  |  |  |  |  |  |
|  |  |  |  |  |  |  |
|  |  |  |  |  |  |  |
|  |  |  |  |  |  |  |
|  |  |  |  |  |  |  |
|  |  |  |  |  |  |  |
