# Supplementary material for: Influenza vaccination hesitancy in large urban centers in South America. Qualitative analysis of confidence, complacency and convenience across risk groups
Source: PLoS One. 2021 Aug 12;16(8):e0256040. doi: 10.1371/journal.pone.0256040 (PMC8360613; doi:10.1371/journal.pone.0256040)
Supplement: Attachment — Submitted filename: Response to reviewers.docx [file pone.0256040.s002.docx]

**Art. PONE-D-21-09160**

**Influenza vaccination hesitancy in large urban centers in South America. Qualitative analysis of confidence, complacency and convenience across risk groups**

Prof. Anat Gesser-Edelsburg, Ph.D.
Academic Editor
PLOS ONE

We are grateful to the comments provided by the two reviewers, which were most useful to improve our paper. We responded to all of them as shown in the table below.

| **Commentary** | | **Response** |
| --- | --- | --- |
| ***Focus group methodology*** | |  |
| Reviewer 1 | 2. The authors do not provide any explanation of why focus groups where chosen as opposed to, e.g., individual interviews. Especially with potential concerns about health information and social desirability bias, I'm not convinced this was the right choice -- it definitely requires a justification as part of the paper's methods section. | We now provide a more detailed justification for the selection of FG methodology. |
| Reviewer 1 | [3. The paper doesn't provide sufficient information about data collection. It would be common to provide basic demographic information about FG participants. I'd also want to know a bit more about recruitment strategies as well as the design of the focus group discussions. Generally speaking, I would encourage authors to follow reporting guidelines for qualitative research such as COREQ (https://doi.org/10.1093/intqhc/mzm042 )](https://doi.org/10.1093/intqhc/mzm042) | We added information regarding recruitment strategies and focus group design. Table 1 was expanded to provide further information regarding the number of participants by sex and risk group, the recruitment strategies, and the average age and number of children in the case of the mothers' groups. |
| Reviewer 2 | 1. FGD process could have been documented better |  |
| Reviewer 2 | 4. FGD could follow maximum variance sampling instead of homogenous grouping to capture diversity of views | We now further justify how and why we recruited focus group participants based on homogeneous as well as heterogeneous risk-group characteristics, this last related to vaccination status. Table 1 provides more detailed information on focus groups composition. |
| Reviewer 2 | 2. Not clear - whether double note takers were there to cross check the tonality and non verbal cues of the participants | We did not use this focus group technique as we did not consider it relevant to code and analyse tonality and non-verbal cues. |
| ***Data analysis procedures*** | | **Unsubscribe** |
| Reviewer 1 | 1. Data analysis procedures are not described in sufficient detail. The authors merely specify that data were coded thematically and don't provide any details on how exactly themes were identified, how coding with multiple coders was coordinated and how consistency was ensured, etc. Significantly more is needed here. | Greater detail is now provided in the methods section regarding the methods used for the development of codes and data interpretation in relation to the 3C conceptual framework. |
| Reviewer 1 | 4.1 The paper's analysis remains somewhat superficial. It gives a point-by-point discussion of the different themes identified, but does little to relate them to each other or make sense of them in the context of the different participating groups. Tables 2-4 are, on the one hand, useful as summaries, but on the other so detailed as to hide any significant analytic points that could be learned from comparing across different risk groups. | The results section compares findings across risk groups in what we believe is appropriate detail. Eg., in the case of safety, we state that "All groups expressed... that the vaccine is safe ...except pregnant women, informants of all risk groups expressed concerns." In the case of trust in the health system, "Confidence in the information offered by service providers varies across risk groups". However, we have now complemented the analysis highlighting similarities and differences across countries, as requested. Furthermore, in the Discussion section we provide further detail on how the various themes relate across each other, highlighting their implications for vaccine hesitancy. We now provide Figure 1 highlighting key findings along the critical dimensions of confidence and complacency. |
| Reviewer 1 | 4.2 Given the significant effort involved in conducting a five-country study, it's also somewhat disappointing that the authors do not take any advantage of this in systematically comparing across cases. | The data-bases were reanalyzed in order to identify significant differences between countries. At the end of each results subsections a paragraph was included highlighting the most relevant findings considering similarities and differences between countries in the item. Main differences found across countries were addressed in the discussion section and the conclusions were modified accordingly. |
| Reviewer 2 | 5. It could analyze the inter regional variance in perception |  |
| Reviewer 2 | 3. RICE analysis could be done on FGD audio transcript to measure frequency of usage of significant phrases | We appreciate the potential of the RICE analysis suggested, but this outside the scope of the paper. Indeed, our aim as to describe and analyse participants perceptions of vaccine hesitancy, rather than the frequency of such perceptions. |
| Reviewer 1 | 5.2 Authors should consider at a minimum a detailed summary of themes and associated descriptions and excerpts in that case | Tables 2-4 aim to provide a detailed summary of themes. As stated in our response to point 4.1 above, we now provide Figure 1 with a caption that further summarizes key findings and dimensions. |
| Reviewer 2 | 6. Some kind of multidimensional perception mapping schematic could be presented from thematic analysis | The schematic is now provided in Figure 1. |
| ***Data availability*** | |  |
| Reviewer 1 | 5.1 The data availability statement is insufficient as per the PLOS data policy. If data can be shared, it should be deposited in a data repository, not per request from the lead author. If the ethics protocol does not allow data sharing (which would be understandable given the nature of the data), this needs to be clearly stated- | Data is now being uploaded to a data repository. The data availability statement will show which repository we use. |
