# Supplementary material for: Influenza vaccination hesitancy in large urban centers in South America. Qualitative analysis of confidence, complacency and convenience across risk groups
Source: PLoS One. 2021 Aug 12;16(8):e0256040. doi: 10.1371/journal.pone.0256040 (PMC8360613; doi:10.1371/journal.pone.0256040)
Supplement: Attachment — Submitted filename: Response to reviewers 06 18.docx [file pone.0256040.s003.docx]

The authors are grateful to reviewers for their useful comments, which we have addressed in our new version.

- We further justify the use of focus group methodology as against alternatives, considering the advantages and limitations
- In the discussion section we address possible limitations of the focus group technique
- We have further detailed the coding methodology
- We added a table providing comparative data on influenza immunization rates by risk group across the five study countries and complemented our discussion with reference to said table
- Reviewers had encouraged us make focus group transcripts available through a data repository. In our previous response we had committed to comply with this recommendation. However, we have now to retract from this commitment given that our consent forms stated that data would be confidential: “All the information you provide us will be kept confidential and we will make this request to all participants. It will be used only by the project researchers and will not be available for any other purpose other than this research.” We are thus unable to publish the transcripts –even when anonymized. We will appreciate the understanding of this.
